# Proinflammatory Microenvironment in Adenocarcinoma Tissue of Colorectal Carcinoma

**DOI:** 10.3390/ijms251810062

**Published:** 2024-09-19

**Authors:** Slobodan Todorović, Miljan S. Ćeranić, Borislav Tošković, Miloš Diklić, Olivera Mitrović Ajtić, Tijana Subotički, Milica Vukotić, Teodora Dragojević, Emilija Živković, Svetlana Oprić, Miodrag Stojiljkovic, Jasna Gačić, Nataša Čolaković, Bogdan Crnokrak, Vladan P. Čokić, Dragoslava Đikić

**Affiliations:** 1 University Hospital Medical Center Bežanijska Kosa, Faculty of Medicine, University of Belgrade, Dr. Žorža Matea bb, 11080 Belgrade, Serbia; bobtod2003@yahoo.com (S.T.); toskeb@gmail.com (B.T.); lana.opric@gmail.com (S.O.); miodrag.stojiljkovic.ms@gmail.com (M.S.); jasna.gacic37@gmail.com (J.G.); colakovicnatasa@yahoo.com (N.Č.); bcrnokrak@yahoo.com (B.C.); 2 University Clinical Center of Serbia, Faculty of Medicine, University of Belgrade, Pasterova 2, 11000 Belgrade, Serbia; miljanceranic1972@gmail.com; 3 Faculty of Medicine, University of Belgrade, Dr. Subotića starijeg 8, 11000 Belgrade, Serbia; 4 Institute for Medical Research, National Institute of Republic of Serbia, University of Belgrade, Dr. Subotica 4, 11129 Belgrade, Serbia; milos.diklic@imi.bg.ac.rs (M.D.); oliveram@imi.bg.ac.rs (O.M.A.); tijana@imi.bg.ac.rs (T.S.); milica.tosic@imi.bg.ac.rs (M.V.); teodora.dragojevic@imi.bg.ac.rs (T.D.); emilija.zivkovic@imi.bg.ac.rs (E.Ž.); vl@imi.bg.ac.rs (V.P.Č.)

**Keywords:** colorectal cancer, adenocarcinoma, inflammation, proliferation, oxidative stress

## Abstract

Cancer-promoting proinflammatory microenvironment influences colorectal cancer (CRC) development. We examined the biomarkers of inflammation, intestinal differentiation, and DNA activity correlated with the clinical parameters to observe progression and prognosis in the adenocarcinoma subtype of CRC. Their immunohistology, immunoblotting, and RT-PCR analyses were performed in the adenocarcinoma and neighboring healthy tissues of 64 patients with CRC after routine colorectal surgery. Proinflammatory nuclear factor kappa B (NFκB) signaling as well as interleukin 6 (IL-6) and S100 protein levels were upregulated in adenocarcinoma compared with nearby healthy colon tissue. In contrast to nitrotyrosine expression, the oxidative stress marker 8-Hydroxy-2′-deoxyguanosine (8-OHdG) was increased in adenocarcinoma tissue. Biomarkers of intestinal differentiation β-catenin and mucin 2 (MUC2) were inversely regulated, with the former upregulated in adenocarcinoma tissue and positively correlated with tumor marker CA19-9. Downregulation of MUC2 expression correlated with the increased 2-year survival rate of patients with CRC. Proliferation-related mammalian target of rapamycin (mTOR) signaling was activated, and Ki67 frequency was three-fold augmented in positive correlation with metastasis and cancer stage, respectively. Conclusion: We demonstrated a parallel induction of oxidative stress and inflammation biomarkers in adenocarcinoma tissue that was not reflected in the neighboring healthy colon tissue of CRC. The expansiveness of colorectal adenocarcinoma was confirmed by irregular intestinal differentiation and elevated proliferation biomarkers, predominantly Ki67. The origin of the linked inflammatory factors was in adenocarcinoma tissue, with an accompanying systemic immune response.

## 1. Introduction

Adenocarcinomas of the colon and rectum account for 96% of all colorectal cancers (CRCs) [1,2]. Colon cancer is a multi-factorial disease that was the second leading cause of cancer death in the EU in 2022 [3]. Most CRCs are sporadic, and the pathogenesis involves genetic and external factors such as deregulation of the Wnt/β-catenin signaling pathway [4]. The nuclear accumulation of β-catenin in epithelial cells initiates the expression of genes involved in cell proliferation, differentiation, and migration [5]. β-catenin enhances the D-Glucuronyl C5-epimerase (GLCE) enzymatic activity in human colon carcinoma cell lines [6]. Colon cancer stem cells are identified by the CD133 marker as one of the main regulators for β-catenin signaling and a predictor of CRC prognosis [7]. In contrast to β-catenin, the tumor-suppressive effect of γ-catenin has been observed in CRC [8]. The intestinal epithelium is covered by a thick layer of mucus. The most abundant secreted intestinal mucin produced by colon epithelial goblet cells is mucin 2 (MUC2). Genetic inactivation of the Muc2 gene causes spontaneous development of gastrointestinal tumors [9]. Muc2 deficiency is linked to low chronic inflammation with cancer development in the intestines involving the rectum [10].

There are increasing indications of a link between inflammation and the development of cancer where invasion-promoting factors (matrix metalloproteinases (MMPs) 2 and 9) and nuclear factor kappa B (NFκB)-dependent proinflammatory cytokine interleukin 6 (IL-6) support the malignant phenotype [11]. A high circulating level of IL-6 is associated with a risk of cancer incidence and poor prognosis [12]. High IL-6 expression in the intestinal mucosa induces expression of the S100A9 protein and activation of NFκB signaling in inflammatory bowel disease [13,14]. Moreover, S100A8/A9 induces cell migration and invasion via NFκB activation with an increase in MMP2 in gastric cancer [15]. S100A8/A9 binds to the receptor for advanced glycation end products (RAGE), leading to inflammation-associated colon carcinogenesis [16]. In addition, S100A8/9 stimulates the mammalian target of the rapamycin (mTOR) signaling pathway that is mediated by RAGE [17].

Recent reports have emphasized the crosstalk between oxidative stress and inflammation in pathological processes. Activation of inflammatory cells results in the production of reactive oxygen and nitrogen species, which can change the structure of macromolecules (DNA, proteins, lipids) [18]. Oxidative/nitrosative stress alters cell signaling to promote cell proliferation and malignant transformation [19]. 8-hydroxy-2′-deoxyguanosine (8-OHdG) is a biomarker of oxidative damage to DNA and a factor of induction and progression of cancer [20].

β-catenin is a functional effector protein of Wnt signaling that plays a central role in the development and progression of CRC. Oxidative stress can activate Wnt/β-catenin signaling, linked to the presented molecules and pathways, with consequent stimulation of the inflammatory cytokines. To evaluate these close connections between oxidative stress, chronic inflammation, and cancer, we observed the proposed and linked biomarkers, which have pathological and prognostic significance, in colorectal adenocarcinoma. In addition to the laboratory and clinical parameters before surgical intervention and the subsequent clinical follow-up related to survival and metastasis, we examined the associated gene and protein expression in adenocarcinoma and nearby healthy tissues by qPCR, immunoblotting, and immunohistochemical analysis. We demonstrated the correlation of the clinical and laboratory parameters with the biomarkers of differentiation, proliferation, and inflammation in patients with colorectal adenocarcinoma.

## 2. Results

### 2.1. Markers of Intestinal Differentiation in Colorectal Adenocarcinoma

The complete blood count test and biochemical parameters of patients with colorectal adenocarcinoma are presented in Table 1. The tumor marker carcinoembryonic antigen (CEA) was generally increased, as were alkaline phosphatase (ALP), creatinine, and C-reactive protein (CRP), in patients with colorectal adenocarcinoma. The neutrophil-to-lymphocyte ratio (NLR, 3.57 ± 2.19) and platelet–lymphocyte ratio (PLR, 216.93 ± 89.07) were also increased in adenocarcinoma.

**Figure 1:**
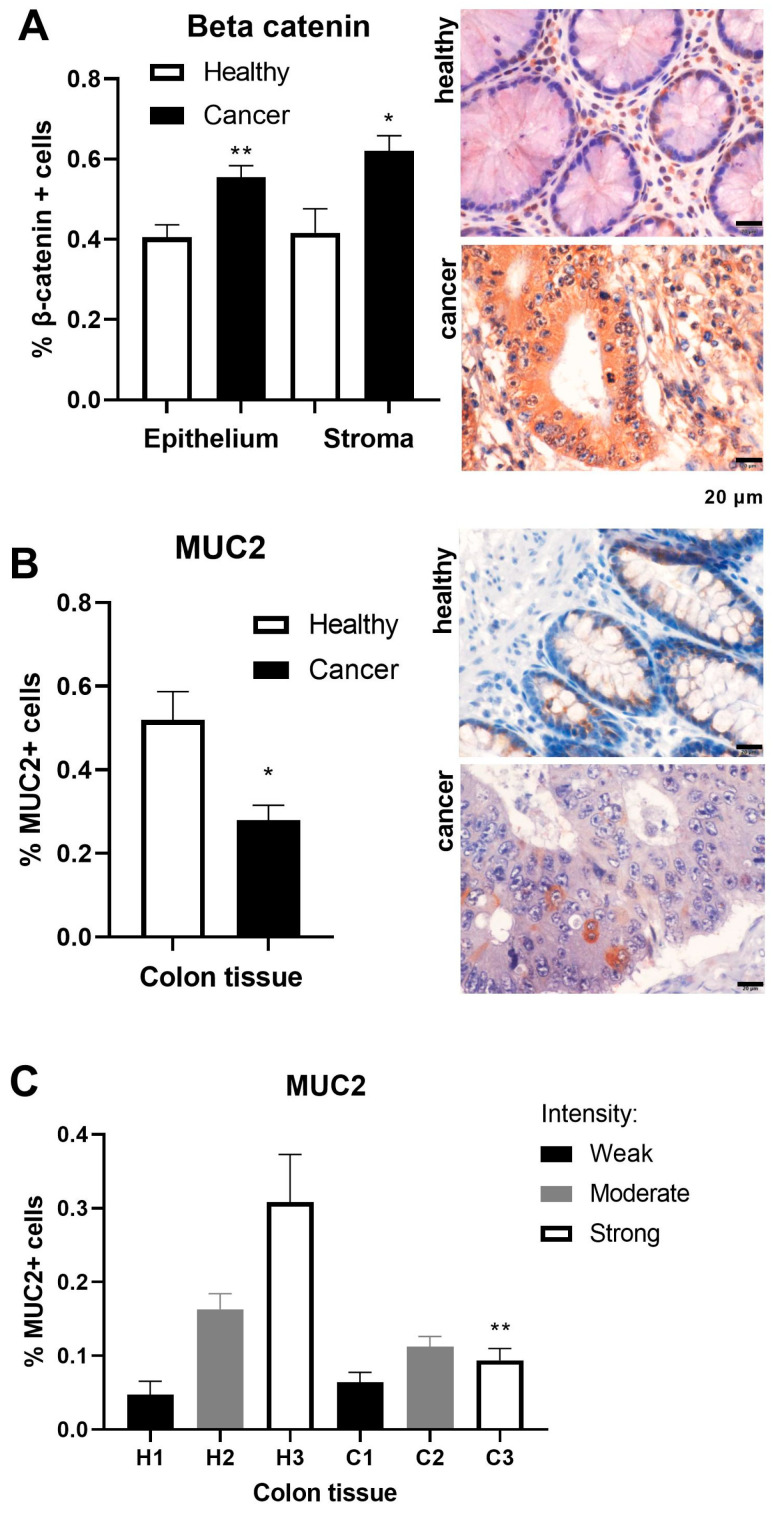
Markers of intestinal differentiation in healthy and adenocarcinoma tissues of the colon. The immunohistochemistry images correspond to the results of antigen frequency of (**A**) β-catenin in healthy (n = 4) and adenocarcinoma (n = 12) tissues of the epithelium and stroma of the colon/rectum; (**B**) mucin 2 (MUC2) in healthy (n = 4) and adenocarcinoma (n = 20) tissues of the colon/rectum; and (**C**) MUC2 in healthy (H1–H3, n = 4) and adenocarcinoma (C1–C3, n = 20) tissues of the colon/rectum. The scale bars in the lower right corner of the microscopic images correspond to a size of 20 μm. The values are the mean ± SEM. * *p* < 0.05, ** *p* < 0.01 vs. healthy tissue.

To examine intestinal differentiation in colorectal adenocarcinoma, we used markers β-catenin and MUC2. Stromal cells may play a significant role by providing a supportive microenvironment. β-catenin frequency was increased both in the epithelium (*p* < 0.01) and stromal (*p* < 0.05) cells of the colon adenocarcinoma (Figure 1A). β-catenin in the stroma was in negative correlation with the level of invasion of regional lymph nodes (Spearman, r = −0.6236, *p* = 0.0336). MUC2 forms an insoluble mucous barrier that protects the colon lumen [21]. MUC2 has a reduced amount of antigen-positive cells in the adenocarcinoma tissue of the colon (Figure 1B). Immunohistochemical staining demonstrated statistical significance (*p* < 0.01) only for the strong intensity of the MUC2 antigen in the adenocarcinoma tissue of the colon (Figure 1C). β-catenin and MUC2 showed inverse expression in the adenocarcinoma tissue of CRC.

### 2.2. DNA Susceptibility and Activity in Colorectal Adenocarcinoma

**Figure 2:**
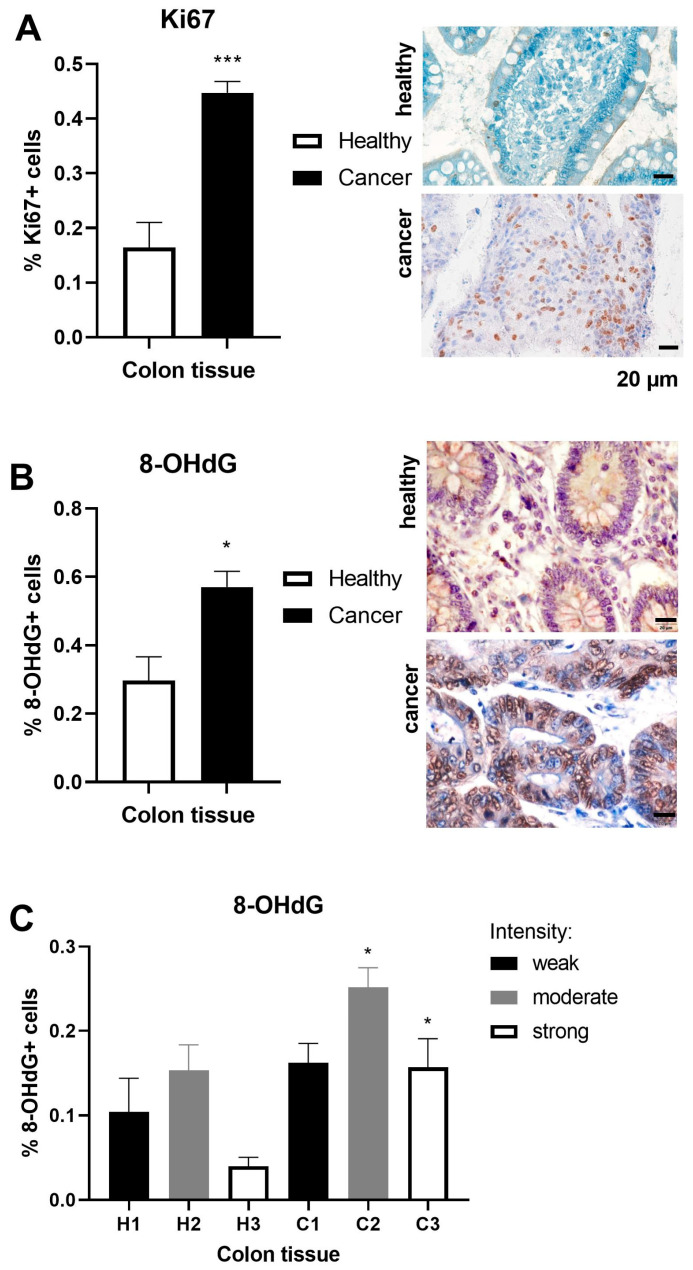
DNA susceptibility and activity in healthy and adenocarcinoma tissues of the colon. The immunohistochemistry images correspond to the results of antigen frequency of (**A**) the proliferation marker Ki67 in healthy (n = 6) and adenocarcinoma (n = 16) tissues of the colon/rectum; (**B**) the marker of oxidative DNA damage 8-hydroxydeoxyguanosine (8-OHdG) in healthy (n = 4) and adenocarcinoma (n = 10) tissues of the colon/rectum; and (**C**) 8-OHdG in healthy (H1–H3) and adenocarcinoma (C1–C3) tissues of the colon/rectum. The scale bars in the lower right corner of the microscopic images correspond to a size of 20 μm. The values are the mean ± SEM. * *p* < 0.05, *** *p* < 0.001 vs. healthy tissue.

8-Hydroxy-2′-deoxyguanosine (8-OHdG) is a product of oxidatively damaged DNA, while proliferation biomarker Ki67 is a prognostic marker for cancers [22]. Ki67 frequency was enlarged three-fold in the adenocarcinoma tissue of the colon (*p* < 0.001, Figure 2A). 8-OHdG frequency was doubled in the adenocarcinoma tissue of the colon compared to healthy tissue (*p* < 0.05, Figure 2B). Immunohistochemical staining demonstrated significance (*p* < 0.05) both for the moderate and strong intensity of the 8-OHdG antigen in the adenocarcinoma tissue of the colon (Figure 2C).

### 2.3. Inflammation Markers in Colorectal Adenocarcinoma

**Figure 3:**
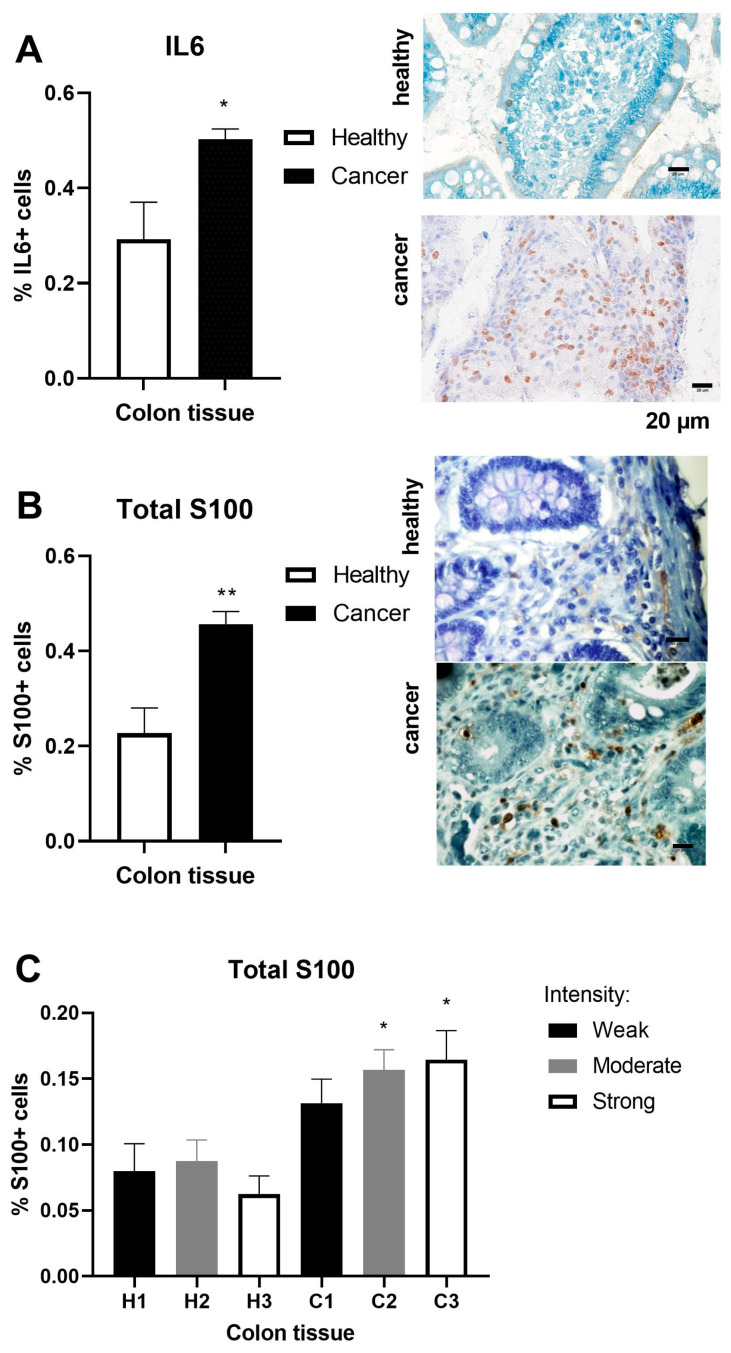
Inflammation markers in healthy and adenocarcinoma tissues of the colon. The immunohistochemistry images correspond to the results of antigen frequency of (**A**) IL-6 in healthy (n = 4) and adenocarcinoma (n = 13) tissues of the colon/rectum; (**B**) S100 in healthy (n = 4) and adenocarcinoma (n = 13) tissues of the colon/rectum; and (**C**) S100 in healthy (H1–H3, n = 4) and adenocarcinoma (C1–C3, n = 13) tissues of the colon/rectum. The scale bars in the lower right corner of the microscopic images correspond to a size of 20 μm. The values are the mean ± SEM. * *p* < 0.05, ** *p* < 0.01 vs. healthy tissue.

Regarding inflammation markers, we examined the frequency of proinflammatory IL-6 and S100 in the adenocarcinoma tissue of the colon. IL-6 frequency was increased in the adenocarcinoma tissue of the colon (*p* < 0.05, Figure 3A). S100 frequency was doubled in the adenocarcinoma tissue of the colon compared to healthy tissue (*p* < 0.01, Figure 3B). Immunohistochemical staining demonstrated significance (*p* < 0.05) both for the moderate and strong intensity of the S100 antigen in the adenocarcinoma tissue of the colon (Figure 3C).

### 2.4. Inflammation and Cancer-Related Gene Expression Profile in Colorectal Adenocarcinoma

**Figure 4:**
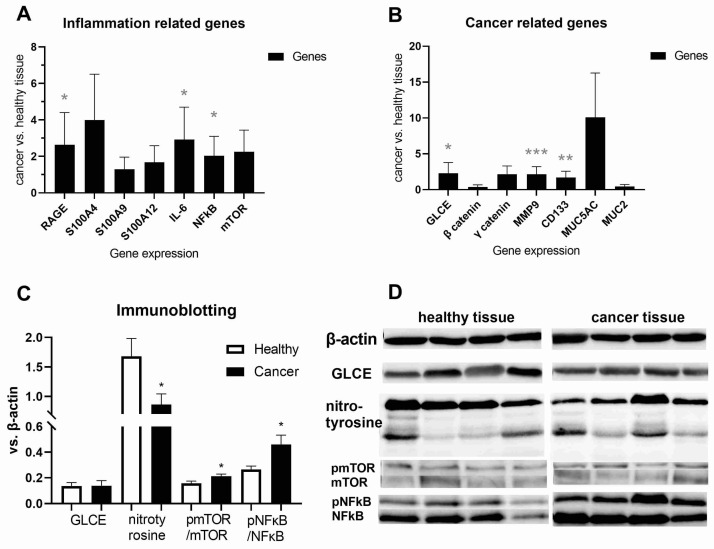
Inflammation- and cancer-related gene expression profiles in cancer and nearby healthy tissues of colorectal adenocarcinoma. qPCR detection of mRNA levels of (**A**) inflammation-related genes: receptor for advanced glycation end products (RAGE), S100A4, S100A9, S100A12, IL-6, nuclear factor kappa B (NFκB), and mammalian target of rapamycin (mTOR) in healthy (n = 10) and adenocarcinoma (n = 10) tissues of colon/rectum; (**B**) cancer-related genes: D-Glucuronyl C5-epimerase (GLCE), β- and γ-catenin, matrix metallopeptidase 9 (MMP-9), CD133, Mucin-5AC (MUC5AC), and MUC2 in healthy (n = 10) and adenocarcinoma (n = 10) tissues of colon/rectum; and (**C**) immunoblotting detection of protein levels of GLCE (n = 30), nitrotyrosine (n = 18), phospho mTOR (pmTOR)/mTOR ratio (n = 22), and pNFκB/NFκB ratio (n = 14) in healthy and cancer tissues of colon/rectum of patients with colorectal adenocarcinoma. (**D**) Densitometry revealed protein expression, as determined by immunoblotting, and is presented as a ratio to β-actin vs. total protein levels or phosphorylated vs. total proteins. Values are mean ± SEM. * *p* < 0.05, ** *p* < 0.01 *** *p* < 0.001 vs. healthy tissue from same patient with colorectal adenocarcinoma.

To observe the expression profile of genes associated with inflammation and cancer at the mRNA level, we performed analyses of adenocarcinoma tissue and nearby healthy colon tissue from the same colorectal adenocarcinoma patient. The qPCR analyses demonstrated increased inflammatory IL-6, the receptor for advanced glycation end products (RAGE), and NFκB gene expression in the adenocarcinoma tissue compared to the bordering healthy tissue of the colon (*p* < 0.05, Figure 4A). However, no increased S100A gene expression was observed with upregulated antigen frequency in the adenocarcinoma tissue (Figure 4A). Potential tumor-suppressor gene GLCE (*p* < 0.05), tumor-associated tissue-remodeling gene MMP9 (*p* < 0.001), and differentiation suppressor CD133 (*p* < 0.01) showed increased gene expression in the adenocarcinoma tissue compared to the neighboring healthy colon tissue (Figure 4B). However, this was not observed for β-catenin and MUC2 gene expression. As an indicator of cell damage and inflammation, protein modification by nitrotyrosine was reduced in the adenocarcinoma tissue compared to the nearby healthy colon tissue (*p* < 0.05, Figure 4C). In contrast, phosphorylation of mTOR and NFκB signaling were increased in the adenocarcinoma tissue of CRC (*p* < 0.05, Figure 4C). The pNFκB/NFκB ratio was doubled in the adenocarcinoma of the rectosigmoideal colon (0.61) compared to the rest of the colon (0.34, *p* < 0.05, Mann–Whitney test).

### 2.5. Correlation of Clinical and Laboratory Parameters with the Markers of Intestinal Differentiation, Proliferation, and Inflammation in Patients with Colorectal Adenocarcinoma

GLCE is an enzyme in the heparan sulfate/heparin synthesis that regulates inflammatory response and tumor metastasis [23]. In addition, GLCE suppresses the proliferation of human breast cancer cells [24]. It was in positive correlation with the age and creatinine levels of patients with colorectal adenocarcinoma (Table 2). Besides a role in cell adhesion, β-catenin has a key role in the regulation of cell proliferation through the Wnt signaling pathway [5]. β-catenin was in positive correlation with tumor marker CA19-9, white blood cells (WBCs), and neutrophils (Table 2). Inflammatory protein S100 was in negative correlation with red cell distribution width (RDW-CV), lymphocytes, and chlorine. MUC2 was in negative correlation with mean corpuscular hemoglobin (MCH) and mean corpuscular hemoglobin concentration (MCHC), and in positive correlation with RDW-CV. Proliferation marker Ki67 was in positive correlation with cancer stage and CRP but in negative correlation with red blood cells (RBCs), hematocrit, hemoglobin, MCHC, and alanine transaminase (ALT, Table 2). mTOR regulates cell proliferation and has also been associated with cancers [25]. Phosphorylated mTOR was positively correlated with plateletcrit (PCT), sodium, and metastasis of colorectal adenocarcinoma (Table 2). Nitrotyrosine was in positive correlation with aspartate aminotransferase (AST). Activated NFκB signaling was negatively correlated with RCB, HGB, HCT, MCHC, and GLCE (Spearman’s r = −0.699, *p* = 0.014) but positively correlated with RDW-CV and PCT (Table 2). According to the number of months after laparoscopy/laparotomy surgery, the general survival rate of patients with colorectal adenocarcinoma was in negative correlation with the S100 protein frequency in adenocarcinoma tissue (Spearman, r = −0.7174, *p* = 0.0362). Moreover, MUC2 frequency was negatively correlated with the 2-year survival rate of the same patients (Spearman, r = −0.4735, *p* = 0.0349). Regarding the total number of CRC patients, the 2-year survival rate was 79.9%, while the 5-year survival rate was 54.7%.

Regarding the TNM classification of malignant tumors, tumor expansion (T0–T3 values) was positively correlated with histopathological grade (Spearman, r = 0.2659, *p* = 0.0418), cancer stage (r = 0.6111, *p* < 0.0001), RDW (r = 0.2495, *p* = 0.0487), and PLT (r = 0.2654, *p* = 0.0355), but in negative correlation with MCV (r = −0.383, *p* = 0.0019) and MCH (r = −0.3626, *p* = 0.0035) in the examined colorectal adenocarcinoma. Regional lymph nodes (N0–N3) were also in positive correlation with histopathological grade (Spearman, r = 0.2616, *p* = 0.0454), cancer stage (r = 0.7155, *p* < 0.0001), and RDW (r = 0.3284, *p* = 0.0086), but in negative correlation with MCV (r = −0.3589, *p* = 0.0039), MCH (r = −0.3849, *p* = 0.0018), and MCHC (r = −0.29, *p* = 0.0211) in the examined adenocarcinoma. Moreover, N values were in positive correlation with AST (r = 0.4465, *p* = 0.0327), ALT (r = 0.4416, *p* = 0.0307), and ALP (r = 0.4633, *p* = 0.0396). According to TNM classification, tumor expansion and regional lymph nodes were in positive correlation with histopathological grade and cancer stage, but not metastases.

## 3. Discussion

More than 90% of CRCs are adenocarcinomas from epithelial cells of the colorectal mucosa, and most colorectal adenocarcinomas (~70%) are diagnosed as moderately differentiated [26]. Cancer-associated systemic inflammation is characterized by a rapid increase in the production of acute-phase proteins CRP, NLR, and PLR, represented in CRC [27]. Inflammatory bowel disease increased the incidence of CRC up to 60% more than in the general population [28]. IL-6 is transcriptionally elevated in colon cancer tissue and is associated with the risk of relapse [29]. NFκB signaling induces inflammation in cancer and targets IL-6 downstream [30]. We demonstrated a stronger activation of NFκB signaling in the adenocarcinoma tissue of CRC compared to healthy colon tissue. We also confirmed increased expression of the IL-6 and NFκB genes, as well as upregulated IL-6 antigen and NFκB signaling in adenocarcinoma tissue. It has been reported that cancer-promoting proinflammatory microenvironments influence cancer progression and metastasis [31]. Considering CRC stage and metastasis, we did not find a significant correlation between proinflammatory IL-6 levels and activated NFκB signaling. As a hallmark of inflammatory conditions, myeloid-derived S100A8/A9 interacted with RAGE on colon tumor cells and activated NFκB signaling pathways to promote tumor growth and metastasis in mouse models [32]. We demonstrated increased RAGE gene expression and increased S100A frequency in adenocarcinoma tissue compared to the healthy tissue of CRC. These results further support the involvement of inflammation in the development of colorectal adenocarcinoma. 

Proliferation-associated mTOR was found to be overexpressed in CRC patients and associated with poor prognosis [33]. We also showed increased activation of mTOR signaling in the adenocarcinoma tissue of CRC with a strong positive correlation to metastasis. Inflammation can initiate tumorigenesis via DNA damage, while reactive oxygen species can cause DNA damage in intestinal epithelial cells [27]. The levels of nitrotyrosine, 8-OHdG, and IL-6 were significantly higher in the venous blood of CRC patients than in healthy volunteers, with a positive correlation between IL-6 and the histological type of the tumor [34]. In contrast to venous blood, nitrotyrosine, as a parameter of nitrosative stress, had a reduced level in the adenocarcinoma tissue of CRC. We showed that 8-OHdG, as a product of oxidatively damaged DNA, has increased levels in the adenocarcinoma tissue of CRC. The correspondence between inflammation-induced DNA damage and cell proliferation has been shown to induce cancer-causing mutations [35]. A continuous increase in Ki67 was detected as the CRC progressed from low to high histological grade and stage [36]. High Ki67 levels negatively correlated with downregulated MUC2 expression [37]. According to the presented results, proliferation marker Ki67 had increased levels and was positively correlated with the stage of colorectal adenocarcinoma. Therefore, we confirmed the overlap between DNA damage and cell proliferation in CRC and demonstrated a linkage to the cancer stage.

MUC2 expression was downregulated to 43% of CRC without correlation between tumor stage or site [38]. A lack of MUC2 expression has been shown to be a predictor of adverse outcome, while an achievement of aberrant MUC5AC expression was associated with a positive outcome in CRC [39]. Regarding intestinal differentiation, we confirmed the reduced expression of MUC2 protein in adenocarcinoma tissue. Upregulated during inflammatory bowel disease, MMP9 maintained the mucosal–epithelial integrity and increased proinflammatory cytokines such as IL-6 [40]. We observed increased MMP9 gene expression that inversely correlated with MUC2 expression during the maturation of intestinal mucosal epithelial cells [41]. Furthermore, we observed increased CD133 gene expression, consistent with the report that high expression of CD133 in epithelial cells correlates with lower survival in colon cancer patients [42]. In positive correlation with tumor marker CA19-9, β-catenin levels were increased in the adenocarcinoma tissue of CRC. Aberrant activation of the Wnt/β-catenin pathway is a key growth driver of CRC development [43]. In contrast to β-catenin, the observed intestinal differentiation biomarker MUC2 was negatively correlated with inflammatory markers. These results agree with the report of Mulyawan that cancerous colonic mucosa had lower expression of MUC2 and higher expression of NFκB in CRC [44]. Some limitations of this study are the small number of CRC patients (64) from a single hospital, the reduced quantity of immunocytochemical (up to 20) and immunoblotting (up to 30) analyses, as well as the limited survival follow-up of patients after surgery (up to 68 months, 5-year survival rate for 54.7% of CRC patients). Considering the molecular basis of CRC, we need genetic testing to detect susceptible mutations despite the high genetic heterogeneity. Regarding functional studies, we lack mechanism-of-action studies involving human cell cultures, such as CACO2 and T84 cell lines, as well as in vivo mouse Apc Min/+ models. Future multicenter, genetic, and long-term studies are necessary to support the proposed complexity of CRC initiation and development.

## 4. Materials and Methods

### 4.1. Clinical and Laboratory Data Collection

All patients were consecutively diagnosed and treated at the Department of Surgery, Clinical Hospital Center Bežanijska Kosa, Belgrade, Serbia. We included 64 patients (31 females and 33 males) with adenocarcinoma subtype of CRC who underwent surgery. Adenocarcinoma tissue confirmed by histopathology and proximal/distal nearby (15–20 cm) healthy colon tissue were collected. The localizations of adenocarcinoma were at the cecum (10 patients), ascending colon (7 patients), hepatic flexure (2 patients), splenic flexure (1 patient), transverse colon (2 patients), descending colon (2 patients), sigmoid colon (9 patients), rectosigmoid junction (5 patients), and rectum (26 patients). Histologically, they were defined as grade 1 (well differentiated, 32.8%), grade 2 (moderately differentiated, 65.6%), and grade 3 (poorly differentiated, 1.6%). The examined patients with colorectal adenocarcinoma had the following stages: I (20.3%), IIA (34.4%), IIC (3.1%), IIIA (1.6%), IIIB (28.1%), IIIC (7.8%), IVA (3.1%), and IVC (1.6%). Moreover, 23% of patients progressed with metastasis, the majority in the liver. Data from the time of diagnosis were extracted and retrospectively evaluated from the medical records. These data included demographic characteristics (age, gender), blood counts, biochemical parameters, histological grades, tumor markers, metastases, molecular cytogenetic studies, and clinical follow-ups after surgery (20.3 ± 15.4 months). This study was performed following the Declaration of Helsinki after approval by the Ethics Committee of Clinical Hospital Center “Bežanijska Kosa” (approval number: 572/2) on 10 February 2022. This study was also approved by the Ethics Committee of the Institute for Medical Research, University of Belgrade, with reference EO136/2022. Written informed consent was obtained from all participants.

### 4.2. RT-qPCR

Expression analysis of genes involved in inflammation and tissue remodeling was assessed by RT-qPCR. Total RNA was isolated from 50–100 mg of RNA later-preserved samples (stored at −80 °C) of adenocarcinoma and nearby healthy colon tissue using Trisure™ reagent (Bioline Ltd., London, UK), according to the manufacturer’s protocol. The concentration and integrity of total RNA were assessed using a Nanophotometer P330 (Implen GmbH, München, Germany). Equal amounts of RNA from different samples were transcribed into cDNA using the High-Capacity cDNA Reverse Transcription Kit with RNase Inhibitor (Thermo Fisher, Waltham, MA, USA) according to the manufacturer’s instructions. Quantitative real-time PCR analyses were performed using FastGene 2x IC Green Universal Kit (Nippon Genetics, Düren, Germany) and Magnetic Induction Cycler (Bio Molecular Systems, Upper Coomera, QLD, Australia). PCR reaction conditions included 50 °C for 2 min and 95 °C for 2 min, followed by cycling between a melting temperature of 95 °C for 3 s and an anneal–extension temperature of 60 °C for 30 s, repeated for 40 cycles, followed by melting curve analyses. For obtaining statistical significance, two technical repeats were used for analyses of target genes, and human β-actin was used as a reference gene. Data analysis was performed in Magnetic Induction Cycler software 2.10.5 (Bio molecular systems Pty Ltd., Upper Coomera, QLD, Australia) using the comparative Ct method (2^−ΔΔCt^). Sequences for primers used for analyses were obtained from Origene (Rockville, MD, USA). All primers were synthesized by IDTDNA (Coralville, IA, Australia), and sequences are listed in Table S1.

### 4.3. Immunohistochemical Procedure

We collected 64 adenocarcinoma tissue samples after routine CRC surgery and 7 healthy colon tissue samples for immunohistochemical analyses. Paraffin-embedded healthy and adenocarcinoma tissues, after routine CRC surgery, were deparaffinized in xylene and rehydrated in descending ethanol series. The antigen retrieval was performed in a citrate buffer by heating the slides in a microwave for 20 min. Endogenous peroxidase was blocked with a 3% H_2_O_2_ solution for 10 min. The slides were incubated overnight at 4 °C with primary antibodies MUC2, IL-6 (Novocastra, Leica Biosystems, Newcastle, UK), protein S100, Ki67 (Dako, Glostrup, Denmark), 8-OHdG (Santa Cruz Biotechnology, Dallas, TX, USA), and β-catenin (NeoMarkers, Fremont, CA, USA). After incubation with biotinized anti-rabbit immunoglobulins, the cells were treated with a streptavidin conjugated to the horseradish peroxidase (UltraVision Detection System, HRP, Thermo Scientific, Waltham, MA, USA). Finally, the slides were incubated in a solution of substrate–chromogen (Liquid DAB + Substrate Chromogen System, Dako, Denmark). Mayer’s hematoxylin was used for contrast. For negative controls, the primary antibody was omitted from the above protocol. The slides were analyzed using an Olympus Provis AX70 microscope (Tokyo, Japan). Analysis was performed on 5 fields per section at a magnification of ×40. Staining intensity was defined as 0/negative: not stained; +: weak staining; ++: moderate staining; and +++: intense immunoreactive staining. The percentage of positively stained cells was determined as 1: 0–50% stained cells; 2: 50–90% stained cells; and 3: over 90% stained cells. Evaluation of antigen expression was determined using a semiquantitative immunohistochemical method by evaluating the intensity of staining and the percentage of stained cells relative to the total number of cells counted.

### 4.4. Immunoblotting Procedure

For protein and RNA isolation, sections of adenocarcinoma and healthy colon tissue from CRC patients were homogenized and lysed using TRIsure (Meridian Bioscience, Cincinnati, OH, USA). Equal amounts of protein samples were run on polyacrylamide gels under reducing conditions and transferred to PVDF transfer membranes (Thermo Scientific Pierce, USA). Membranes were probed with primary antibodies to mammalian targets of rapamycin mTOR and NFkB (Cell Signaling Technology, Danvers, MA, USA), pmTOR (Ser2248), nitrotyrosine and β-actin (R&D Systems, Minneapolis, MN, USA), and glucuronic acid epimerase (GLCE, Abcam, Cambridge, UK). Peroxidase-conjugated goat anti-rabbit immunoglobulin (Santa Cruz Biotechnology) and goat anti-mouse immunoglobulin (Thermo Scientific Pierce, Waltham, MA, USA) were used as secondary antibodies. The protein levels were imaged with a ChemiDoc Imaging System (Bio-Rad Laboratories, Hercules, CA, USA) and estimated by densitometric scanning of the blots using the Image Lab (Bio-Rad Laboratories, Inc., Version 6.0.0.25, Hercules, CA, USA) software tool and normalized to β-actin.

### 4.5. Statistical Analysis

The normality of data distribution was examined by Shapiro–Wilk and Kolmogorov–Smirnov tests. Differences between groups were analyzed using Student’s *t*-test. When the distribution was not normal, Mann–Whitney was used for intergroup comparisons. Correlations between numerical variables were assessed by Pearson’s or Spearman’s correlation coefficients. All statistics were performed using Prism 6 software (GraphPad Software Inc., San Diego, CA, USA). Results are expressed as mean ± SEM. The level of significance was 5%.

## 5. Conclusions

Intestinal differentiation was differentially regulated by colonic epithelial cells in CRC, with a reduced insoluble mucosal barrier (marked by MUC2) and elevated adherens junctions between cells (marked by β-catenin). Decreased MUC2 expression was correlated with an increased 2-year survival rate in CRC patients. Since overexpression of β-catenin is associated with CRC, we demonstrated its positive correlation with tumor marker CA19-9. Intensified in adenocarcinoma tissue, proliferation (marked by Ki67) positively correlated with the stage and metastasis of CRC (marked by mTOR). Proinflammatory biomarkers were increased, in parallel with oxidatively damaged DNA, in the adenocarcinoma tissue of CRC. Increased S100 expression in adenocarcinoma tissue was correlated with reduced survival rate in CRC patients. The multistage invasiveness of CRC is regulated by combined deviations in intestinal differentiation, proliferation, and inflammation. This complex approach is responsible for the progress and metastasis of CRC.

## Figures and Tables

**Table 1:** The complete blood count test and biochemical parameters of patients with colorectal adenocarcinoma.

Blood Elements	AV	SD	Normal Values	Biochemical Parameters	AV	SD	Normal Values
CEA (ng/mL)	9.08	20.91	<2.5	Urea (mmol/L)	6.98	7.97	2.8–8.1
CA19-9 (U/mL)	13.79	12.97	<37	Glucose (mmol/L)	6.59	2.82	4.1–5.9
WBC (10^9^/L)	7.37	2.68	4.0–10.0	Bilirubin direct (μmol/L)	2.54	1.04	<6.0
RBC (10^12^/L)	4.30	0.61	4.5–6.5	Bilirubin total (μmol/L)	11.13	5.88	<21.0
HGB (g/L)	116.8	23.33	130–170	AST (U/L)	22.87	13.20	<32.0
HCT (%)	36	6	39–50	ALT (U/L)	25.88	28.76	<33.0
MCV (fl)	83.74	8.28	82–99	ALP (U/L)	141.5	182.4	35–105
MCH (pg)	27.17	3.70	27–32	LDH (U/L)	291.8	114.1	270–428
MCHC (g/L)	323.4	16.77	320–360	K (mmol/L)	4.45	0.59	3.5–5.1
RDW-CV (%)	15.57	3.11	12–15	Na (mmol/L)	139.6	3.45	136–145
PLT (10^9^/L)	298.8	85.70	150–450	Cl (mmol/L)	100.5	4.45	98–107
PCT %	0.28	0.08	0.16–0.35	Ca (mmol/L)	2.39	0.17	2.1–2.6
NEUT %	66.13	9.61	44–77	Mg (mmol/L)	0.78	0.08	0.66–1.07
LYM %	22.34	7.89	20–46	Phosphorus (mmol/L)	1.04	0.15	0.8–1.5
NEUT (10^9^/L)	4.96	2.30	2.0–7.5	Creatinine (μmol/L)	116.9	134	44–80
LYM (10^9^/L)	1.58	0.69	1.0–4.0	Amylase (mmol/L)	74.57	28.31	28–100
Age (years)	67.38	9.84		Protein (g/L)	66.55	10.36	64–83
Body mass (kg)	75.04	15.63		Albumin (g/L)	38.88	5.99	35–52
				CRP (g/L)	33.77	50.46	<5.0

WBC—white blood cell; RBC—red blood cell; HGB—hemoglobin; HCT—hematocrit; MCV—mean corpuscular volume; MCH—mean corpuscular hemoglobin; MCHC—mean corpuscular hemoglobin concentration; RDW-CV—red blood cell distribution width; PLT—platelet blood cell; PCT—plateletcrit; NEUT—neutrophils; LYM—lymphocytes; AST—aspartate aminotransferase; ALT—alanine aminotransferase; ALP—alkaline phosphatase; LDH—lactate dehydrogenase; CRP—C-reactive protein.

**Table 2:** Correlation of clinical and laboratory parameters with markers of intestinal differentiation, proliferation, and inflammation in patients with colorectal adenocarcinoma.

Clinical and Laboratory Parameters		β-Catenin in Epithelium and Stroma	Ki67 S100	GLCE MUC2	pmTOR/mTOR Nitrotyrosine	pNFκB/pNFκB
Age	r			0.690		
	p			<0.0001		
CA19-9 (U/mL)	r	0.837				
	p	0.006				
Metastasis	r				0.592	
	p				0.004	
Cancer stage **	r *		0.623			
	p		0.017			
WBC (10^9^/L)	r	**0.621**				
	p	**0.027**				
RBC (10^12^/L)	r		−0.624			−0.703
	p		0.015			0.009
HCT (%)	r *		−0.670			−0.731
	p		0.006			0.006
HGB (g/L)	r *		−0.669			−0.661
	p		0.006			0.016
MCH (pg)	r *			**−0.507**		
	p			**0.027**		
MCHC (g/L)	r *		−0.541	**−0.620**		−0.685
	p		0.038	**0.005**		0.012
RDW-CV (%)	r		**−0.786**	**0.541**		0.613
	p		**0.028**	**0.017**		0.0287
PCT (%)	r				0.462	0.636
	p				0.030	0.022
Lymphocyte (%)	r *		**−0.734**			
	p		**0.038**			
Neutrophile (10^9^/L)	r	**0.769**				
	p	**0.003**				
ALT (U/L)	r		−0.870			
	p		0.033			
AST (U/L)	r				**0.975**	
	p				**0.033**	
Na (mmol/L)	r *				0.975	
	p				0.033	
Cl (mmol/L)	r *		**−0.998**			
	p		**0.039**			
Creatinine (μmol/L)	r			0.685		
	p			0.035		
CRP (g/L)	r		0.975			
	p		0.033			

* Pearson’s r (the rest are Spearman’s r). ** ranged from stages I through IV. Red cell distribution width—RDW-CV; D-Glucuronyl C5-epimerase—GLCE; mucin 2—MUC2. Other abbreviations are presented in the footnote of Table 1. Non-bolded and bolded values correspond to the first and second marker in the head row of Table 2, respectively.

## Data Availability

The original contributions presented in this study are included in the Supplementary Materials; further inquiries can be directed to the corresponding author. The raw data supporting the conclusions of this article will be made available by the authors upon request.

## Supplementary Materials

The following supporting information can be downloaded at https://www.mdpi.com/article/10.3390/ijms251810062/s1.
