# Supplementary material for: Proinflammatory Microenvironment in Adenocarcinoma Tissue of Colorectal Carcinoma
Source: Int J Mol Sci. 2024 Sep 19;25(18):10062. doi: 10.3390/ijms251810062 (PMC11432548; doi:10.3390/ijms251810062)
Supplement: Supplementary file 1 [file ijms-25-10062-s001.zip › ijms-3190502-supplementary.pdf]

Supplemental Table S1. Primers of genes involved in inflammation and tissue remodeling.

Genes	Primers	Sequence of primers
MUC2	forward	ACTCTCCACACCCAGCATCATC
	reverse	GTGTCTCCGTATGTGCCGTTGT
MUC5AC	forward	CCACTGGTTCTATGGCAACACC
	reverse	GCCGAAGTCCAGGCTGTGCG
β-catenin	forward	CACAAGCAGAGTGCTGAAGGTG
	reverse	GATTCCTGAGAGTCCAAAGACAG
γ-catenin	forward	ACCAGCATCCTGCACAACCTCT
	reverse	GGTGATGGCATAGAACAGGACC
IL-6	forward	AGACAGCCACTCACCTCTTCAG
	reverse	TTCTGCCAGTGCCTCTTTGCTG
p53	forward	CCTCAGCATCTTATCCGAGTGG
	reverse	TGGATGGTGGTACAGTCAGAGC
CD133	forward	CACTACCAAGGACAAGGCGTTC
	reverse	CAACGCCTCTTTGGTCTCCTTG
MMP9	forward	GCCACTACTGTGCCTTTGAGTC
	reverse	CCCTCAGAGAATCGCCAGTACT
mTOR	forward	AGCATCGGATGCTTAGGAGTGG
	reverse	CAGCCAGTCATCTTTGGAGACC
NFκB1	forward	GCAGCACTACTTCTTGACCACC
	reverse	TCTGCTCCTGAGCATTGACGTC
GLCE	forward	TTCCAAAGTCTATGCACAGAGAGC
	reverse	TCCACATTGTAGCCTTCAAAAGACA
S100A4	forward	CAGAACTAAAGGAGCTGCTGACC
	reverse	CTTGGAAGTCCACCTCGTTGTC
S100A9	forward	GCACCCAGACACCCTGAACCA
	reverse	TGTGTCCAGGTCCTCCATGATG

S100A12	forward	CTCTAAGGGTGAGCTGAAGCAG
	reverse	ACCTGTTCATCTTGATTAGCATCC
